# Influence of Biological Sex and Congenital Iron Deficiency on Neonatal Cytokine Responses

**DOI:** 10.3390/nu16234203

**Published:** 2024-12-05

**Authors:** Narmin Mukhtarova, Anthony Babu, Christopher L. Coe, Pamela J. Kling

**Affiliations:** 1 Department of Pediatrics, University of Wisconsin Hospitals and Clinics, Madison, WI 53792, USA; mukhtarova@wisc.edu; 2 Department of Pediatrics, University of Wisconsin-Madison, Madison, WI 53792, USA; anthonyjbabu@gmail.com; 3 Department of Psychology, University of Wisconsin-Madison, Madison, WI 53706, USA; ccoe@wisc.edu

**Keywords:** cord blood, interleukins, cytokines, nutrition, iron, atopy, inflammation

## Abstract

Background/Objectives: Stimulated cord blood mononuclear cell (CBMC) cytokine responses were previously shown to predict the risk of childhood atopic disease. Iron deficiency (ID) at birth may also program atopic disease. Males are at a higher risk of pediatric atopic disease, but it is not known whether congenital ID impacts CBMC immune responses differentially by sex. Methods: Cord blood (CB) samples were collected from healthy term or near-term neonates after elective cesarean deliveries. A transferrin saturation ≤ 25% defined congenital ID. CBMCs were stimulated with either phytohemagglutinin (PHA) or PHA plus an iron chelator. Results: Of the 85 neonates, the 26 neonates with congenital ID exhibited lower plasma tumor necrosis factor-α (TNF-α), as well as higher CBMC TNF-α and IL-8 responses than iron-sufficient neonates (*p* = 0.017, *p* = 0.013, and *p* = 0.007, respectively). Higher CBMC TNF-α responses were seen in both males and females with congenital ID. However, females with congenital ID also had lower plasma IL-6, lower plasma TNF-α, and higher CBMC interleukin (IL)-8 responses. Additionally, iron chelation during culture influenced stimulated CBMC IFN-γ and CBMC TNF-α responses. Discussion: Congenital ID may influence stimulated CBMC cytokine responses, but results point to a sex-specific regulation of immune balance at birth. Because males are more prone to infantile ID and more likely to develop early childhood asthma, future studies should further investigate how fetal sex and congenital iron status impacts childhood immune responsiveness to infections and antigenic stimulation from the rearing environment.

## 1. Introduction

Atopic disease is a substantial pediatric concern, making it especially important to identify early modifiable biological and environmental risk factors [1]. Fetal iron status may be one candidate. Iron has many essential functions in fetal cell differentiation and proliferation. Maternal iron deficiency (ID) is extremely common during pregnancy, affecting more that 50% of gravid women in the absence of iron supplementation, and it is one of the major risk factors for neonates being born with insufficient iron reserves, i.e., congenital ID [2]. Maternal or congenital ID may contribute to developing atopic disease during the first year of life. Maternal ID during pregnancy was previously shown to be associated with the risk of wheezing symptoms in children [3]. Further, iron concentrations in umbilical cord blood (CB) were negatively associated with the later prevalence of both infantile wheezing and eczema [4]. Additionally, we previously showed that poor iron status in the CB at delivery predicted infantile eosinophilia, a cellular biomarker for atopic disease [5]. Furthermore, anemia, a hematological consequence of congenital ID, was associated with higher levels of the inflammatory cytokine interleukin-6 (IL-6) in the CB of neonates born in Tanzania [6].

The circulating levels of inflammatory cytokines at birth have been found to predict later atopic disease. High levels of IL-8 in CB plasma predicted higher rates of persistent infantile wheezing [7]. Eliciting cytokine release in vitro in CB mononuclear cells (CBMCs) by mitogen stimulation was also found to predict a propensity for atopy and asthma. For example, tumor necrosis factor-α (TNF-α) responses by both stimulated CBMCs at birth and by peripheral blood mononuclear cells (PBMCs) at 3 months of age predicted higher rates of childhood asthma [8]. Low interferon (IFN)-γ responses by stimulated CBMCs were also associated with greater immune sensitization [9].

Another biological factor known to influence immune responses at birth and during childhood is sex [10]. First, young males typically have less-robust immune responses post-immunization and post-infection compared to females in both humans and animal models [11]. In addition, sex-specific stimulated CBMC and early childhood PBMC responses may contribute to males being more likely to develop atopic dermatitis, allergic sensitization, and/or wheezing [12,13,14]. Because iron status can impact immunity and other cellular processes, the fact that males are more likely to become iron deficient in early infancy could feasibly contribute to the differential sensitivity of male infants to allergens [15]. In addition to the potential impact of iron, sex-specific hormones may influence the maturation of immune responses. For example, higher CB dihydrotestosterone (DHT) levels of male neonates were associated with more immature B lymphocytes at birth, a risk factor for allergies and smaller antibody responses to immunization, and DHT in males was related to a difference in the proportion of regulatory T cells in circulation and the stimulated release of T-cell-related cytokines such as IFN-γ through 8 years of age [16]. Thus, the male-specific perinatal surge of testosterone or its potent metabolite, DHT, may differentially program immune responses [16,17]. Post-puberty, ID becomes more prevalent in adolescent females due to menstruation, and the influence of ovarian hormones on the immune system leads to a higher prevalence of atopic disorders among females [18,19,20,21,22,23].

Historically, Mosmann et al. introduced the concept of a dichotomy in T helper 1 (Th1) cells and Th2 lymphocytes based on synthesized cytokine profiles and actions, with a Th1 bias promoting cellular and cytotoxic responses and a Th2 bias enhancing antibody production [24]. Cytokines were later categorized into different subgroups based on Th1 polarization (e.g., IL-2, IFN-γ, IL-12, and TNF-α,) and Th2 cytokines (e.g., IL-4, IL-5, IL-6, and IL-10). Further, this division suggested that Th2 activation might potentiate an allergic response, whereas neonates who mounted stronger Th1 cytokine responses would not be as predisposed to developing atopic conditions [24]. From the perspective of the current research on iron and immunity, it also important to consider that Th2 lymphocytes have higher intracellular iron stores than do Th1 lymphocytes [25], a finding that could contribute to a differential expansion of thymocyte subsets at birth.

In an earlier paper on the mothers and neonates assessed in our new analysis, we reported that class II or higher obesity in pregnancy was indirectly associated with CB iron storage and directly associated with the cytokine responses of CBMCs stimulated in vitro with PHA [26]. However, our prior analysis did not consider either biological sex or the balance of different cytokines associated with how CBMCs were polarized after being stimulated in the culture and how this should be considered for new pilot analyses. Further, additional new pilot analyses probe how the cytokine release of the cells is affected when the intracellular availability and actions of iron are pharmacologically blocked with deferoxamine. The objective was to extend the research in a novel way by examining the interactions between CB iron status and biological sex on CB plasma and CBMC cytokine profiles. We hypothesized that a poor iron status at birth might be reflected in the stimulated CBMC cytokine profiles, especially for inflammatory mediators like TNF-α that are associated with atopic conditions, and that this modifiable risk might be differentially evident in female and male neonates.

## 2. Materials and Methods

### 2.1. Study Population

CB was collected from 85 healthy neonates, gestational age ≥ 37 weeks, during 2012–2014, as previously described [26]. Collections were scheduled only during the summer months to avoid seasonal variation in allergen and viral exposure, and annual fluctuations in cytokine responses [27]. All births were at UnityPoint Health, Meriter Hospital, Madison, WI, by scheduled elective cesarean delivery to limit the possible impact of natural labor and variation from a delay in sample processing, as well as the inclusion of premature births or inflammation such as chorioamnionitis [26]. Maternal and newborn demographic and anthropometric data were obtained from the electronic medical records. UnityPoint Health—Meriter (IRB) determined that parental consent was not required because identifiers were not retained. In addition to biological sex, data included self-reported risks for congenital ID, including maternal ethnicity and race, insurance, maternal-delivery body mass index (BMI) with obesity defined as BMI > 30 kg/m^2^, maternal diabetes, neonatal sex, and whether the neonate was calculated as being either small-, appropriate-, or large-for-gestational age. We found previously that the risk for congenital ID was higher when ≥3 of these predisposing risk factors were present [28]. Smoking status was not recorded; but, a 9.5% rate was estimated. Exclusion criteria included maternal HIV infection, cancer, chronic hypertension, gestational hypertension, diagnosed chorioamnionitis, multiple medication prescriptions, fetal hemolytic disease, neonatal intensive-care admission, major fetal anomaly, or an estimated fetal weight below the first percentile. Three samples were excluded with extreme stimulated CBMC IFN-γ and TNF-α responses >99th percentile post hoc [26]. These excluded CBMC responses that were remarkably 3- to 10-fold higher than other mean CBMC IFN-γ responses. Because IFN-γ responses are normally blunted in healthy neonates, it was presumed that the IFN-γ and TNF-α pathways were either activated during processing or that there was an underlying inflammatory process in a neonate presumed to be delivered by a scheduled cesarean.

### 2.2. Sample Processing

Heparinized CB was processed within 18 h of collection (median = 2.25 h), with only 2 samples delayed for >8 h. Neither maternal blood nor postnatal infant venous blood samples were collected. Complete blood counts were assayed via a Sysmex PocH-100i hematology analyzer (Sysmex, Mundelein, IL, USA). Plasma assays included ferritin (Genway Biotech, San Diego, CA, USA), transferrin (ICL, Portland, OR, USA), unsaturated iron-binding capacity and iron (Pointe Scientific, Canton, MI, USA), hepcidin (DRG International, Springfield, NJ, USA), C-reactive protein (CRP, Genway Biotech, San Diego, CA, USA), and IL-6 and TNF-α (R&D Systems, Minneapolis, MN, USA). Reticulocyte (Reticulocyte Stain, Sigma–Aldrich, St. Louis, MO, USA) and eosinophil (Wright–Giemsa Stain, Astral Diagnostics, West Deptford, NJ, USA) counts were determined manually, blinded to clinical and other laboratory data.

### 2.3. Stimulated CBMC Responses

After centrifugation and plasma removal, CBMCs were isolated using lymphocyte separation medium (Lonza, Walkersville, MD, USA) and cell counts were determined. Cells were cultured at 0.5 to 1 × 10^6^ cells/mL in complete media prepared from RPMI-1640 with 10% FBS, L-glutamine (2 mM), Pen Strep (100 U/mL) and Hepes (25 mM) [27]. To analyze stimulated cytokine responses, 500 µL of the CBMC suspensions in 500 µL of complete media with and without phytohemagglutinin (PHA) stimulation, 5 µg/mL, were added to 24-well plates and incubated at 37 °C for 24 h. PHA is a plant lectin that serves as a T-lymphocyte-stimulating mitogen. The direct influence of iron availability on the CBMC cytokine response was also determined. In duplicate wells, we depleted iron using iron chelation with deferoxamine (50 µM) to PHA (5 µg/mL), also incubating at 37 °C for 24 h. To examine if the CBMC processing affected cell viability, a thiazolyl blue tetrazolium bromide (MTT) dye colorimetric assay verified viability after incubating with the MTT solution (0.9 mg/mL, Alfa Aesar, Ward Hill, MA, USA) at 37 °C for 4 h, followed by solubilized dimethyl sulfoxide at 200% total volume, read at 570 nm. We compared the MTT absorbance values of PHA plus deferoxamine to PHA alone, finding that values for PHA plus deferoxamine were 94.5% of PHA (CI 88.9–100.0), supporting that the cells remained metabolically active.

After overnight incubation, CBMC supernatants were initially frozen with liquid nitrogen, stored at −80 °C, and assayed later for INF-γ, IL-1β, IL-5, IL-6, IL-8, IL-10, IL-12, and TNF-α using a Human Proinflammatory multi-plex array (Meso Scale Discovery, Rockville, MD, USA). Supernatant IL-5 concentrations were assayed separately via a high-sensitivity ELISA (R&D Systems, Minneapolis, MN, USA). Samples were aliquoted to avoid freeze–thaws and stored at −80 °C. Because cytokine assays are sensitive to prolonged storage and freeze–thaw cycles, samples were collected between June 2012 and July 2014 with all immunoassays performed between January and July 2015, 0.5–3 years before the immunoassay.

### 2.4. Statistical Analysis

CB plasma profiles and stimulated CBMC responses were initially analyzed and stratified based on biological sex. Although ID can be defined by either low erythrocyte iron, storage iron, or transport iron, we defined congenital ID by transferrin saturation (TSAT) because the transferrin–transferrin receptor-mediated transport is the predominant iron delivery route to mononuclear cells. For this study, we defined congenital ID as TSAT < 36%, which is less than the 25th percentile of our data but is approximately the 5th percentile for neonatal normal values as congenital ID, comparing those samples to the iron-sufficient (IS) controls. We evaluated cytokine levels to determine whether they showed an overall proinflammatory bias. Heatmaps of Pearson’s correlation coefficients were used to evaluate relationship patterns between stimulated CBMC cytokine responses. For comparisons and due to differences in the levels of individual cytokines, the pg/mL concentrations were converted to ranks as *Z*-scores ((absolute value—mean)/standard deviation). Because atopic diseases are associated with a bias for higher levels of Th2 cytokines, several cytokines associated with either a Th1 or Th2 polarization were selected and examined as a subgroup. IL-12, TNF-α, and INF-γ were categorized as initiating Th1 polarization; IL-5, IL-6, and IL-10 were categorized as predisposing for a stronger Th2 response. The proinflammatory proteins included 3 cytokines, TNF-α, IL-1β, and IL-6, in the nuclear factor-κβ (NF-κβ)-dependent family, in addition to other NF-κβ family members, IL-8 and IL-12. Because maternal obesity and diabetes have been associated with stimulated cytokine responses previously, we analyzed these, but did not find these to be confounding variables in our study.

The Shapiro–Wilk normality test was used to examine data distribution. If data were either normally distributed or normalized after natural log conversion, Student’s *t* tests or one-way ANOVAs were employed. If data were still skewed, nonparametric Wilcoxon rank-sum or Kruskal–Wallis tests were used. This pilot study was not powered to determine differences in cytokines between ID and sex. We also performed 2-way ANOVA modeling of those cytokine response variables showing significance with both ID and sex, finding significance in CBMC IL-8 (F = 3.19, *p* = 0.031) and TNF-α (F = 3.36, *p* = 0.026), but these did not survive correction for multiple comparisons. The analyses exploring the interaction between ID and sex are exploratory in nature. Statistical analyses were performed with StataSE 17 and GraphPad Prism 9. All statistical tests were two-tailed tests, with the alpha level setting *p* < 0.05.

## 3. Results

### 3.1. Demographic and Clinical Characteristics

This healthy cohort included 85 healthy term neonates. Maternal and neonatal sociodemographic data published previously [26] are shown in Table 1. Most mothers identified as Caucasians. The mean (SD) CB ferritin level was 90.9 (48.5) µg/L, which was below the normal range [28], while the mean (SD) TSAT level was 49.7 (26.6), which was within the normal range [29,30]. Thirty percent were at risk of congenital ID with more than or equal to three established ID risk factors [28].

### 3.2. Congenital ID

The CB iron indices, plasma inflammatory indices, and stimulated CBMC cytokine responses are shown for IS and congenital-ID neonates (i.e., low TSAT) in Table 2. We previously found that CB hemoglobin and mean cell volume were relatively insensitive to changes in fetal iron status and may not fall in the presence of obesity ± diabetes in ID [26]. However, plasma ferritin levels were lower in neonates with congenital ID. Neonates with congenital ID exhibited lower plasma TNF-α levels and higher PHA-stimulated CBMC IL-8 and TNF-α responses than the IS neonates, as shown in Table 2. We then examined PHA-stimulated CBMC cytokine responses after deferoxamine chelation and determined that both CBMC INF-γ and TNF-α responses were lower in the cell supernatants from congenital ID neonates than the from IS neonates, Table 2. It is noteworthy that the striking rise in PHA-stimulated CBMC TNF-α responses in the congenital ID group was also attenuated by iron chelation with deferoxamine, as shown in Table 2. Deferoxamine did not affect CBMC viability. CBMC viability measured by MTT after 24 h of incubation did not differ between PHA stimulation and the PHA plus deferoxamine stimulation (*p* > 0.5).

### 3.3. Relationships Between Cytokines with PHA and PHA Plus Deferoxamine

**Figure 1:**
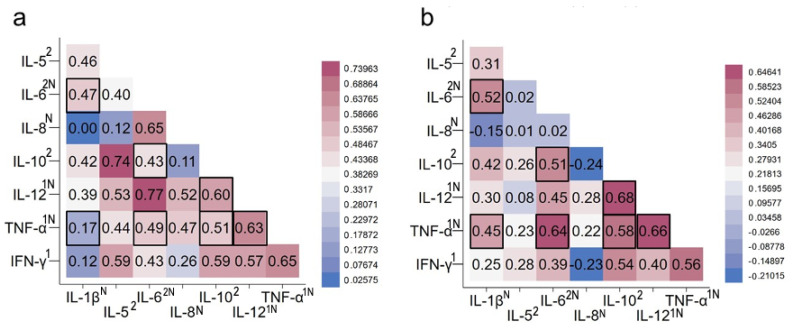
Heatmaps of Pearson’s correlation coefficients in stimulated cytokine responses. (**a**) PHA-stimulated CBMC; (**b**) PHA stimulation co-cultured with deferoxamine. Red boxes indicate positive correlations, blue boxes negative correlations; darker color hues reflect stronger relationships. Vertical column, right, is numerical scale for color code. Highlighted black boxes indicate correlations strengthened after iron chelation with deferoxamine. Superscripts: ^1^ indicates Th1 and ^2^ indicates Th2 categorization of cytokines; ^N^ indicates proinflammatory cytokines in NFκβ family. Abbreviations: PHA, phytohemagglutinin; CBMC, cord blood mononuclear cell; IL, interleukin; TNF-α, tumor necrosis factor alpha; IFN-γ, interferon gamma.

To further examine the impact of iron status on multiple stimulated CBMC cytokine responses, we compared heatmaps of Pearson’s correlation coefficients between different cytokine responses under the (a) PHA- and (b) PHA-plus-deferoxamine-stimulated conditions (Figure 1). Positive and strong correlations were evident between many PHA-stimulated cytokine responses, including the most robust between the two Th2 cytokines IL-5 and IL-10 (R = 0.74) and between a Th2 (IL-6) cytokine and a Th1 (IL12) cytokine (R = 0.77), both of which are NF-κβ-regulated cytokines (Figure 1a). Relationships between TNF-α and other cytokine responses after deferoxamine were most notable. Incubation with deferoxamine strengthened the associations between PHA-stimulated TNF-α responses with four other stimulated cytokines, namely IL-1β, IL-6, IL10, or IL-12 responses. Three cytokines, IL-1β, IL-6, and IL-12, are also NF-κβ-regulated cytokines (Figure 1b). Deferoxamine also strengthened the relationship between PHA-stimulated IL-6 with IL-1β responses and, also the relationship between stimulated IL10 with IL-6 and IL12 responses (Figure 1b).

### 3.4. Comparing Th1 and Th2 Cytokine Z-Scores by Iron and by Sex

**Figure 2:**
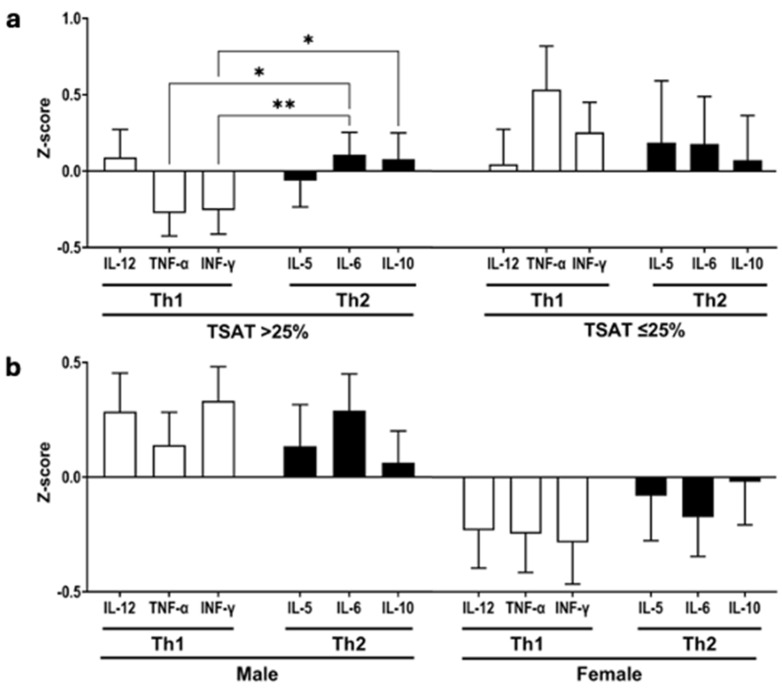
Relationships between stimulated CBMC Th1 and Th2 cytokines. (**a**) Iron-sufficient (IS, TSAT > 25%, means > 25th percentile, with a TSAT value > 36%) and iron-deficient neonates (ID, TSAT ≤ 25%, means ≤ 25th percentile, with a TSAT value ≤ 36%); (**b**) male and female. Bar graph shows mean (SEM) *Z*-score based on rank. White bars are Th1 cytokines; black bars are Th2 cytokines, * *p* < 0.05, ** *p* < 0.01. Abbreviations: TSAT, transferrin saturation; CBMC, cord blood mononuclear cell; Th1, helper T cells polarizing for cellular immune responses; Th2, helper T cell polarizing for humoral immune responses; IL, interleukin, TNF-α, tumor necrosis factor alpha; IFN-γ, interferon gamma.

Because absolute levels of cytokines differ, the concentration values were converted to Z-scores to compare the cytokine profiles from each neonate’s samples. Three cytokines representative of Th1 and Th2 cytokine polarization in the cell supernatants were compared between the IS and congenital ID conditions, as shown in Figure 2a. Stimulated CBMCs from the IS neonates released higher IL-6- and IL-10-response (Th2 cytokines) *Z*-scores relative to their TNF-α and INF-γ responses (Th1 cytokines): * *p* < 0.05, ** *p* < 0.01. IS neonates with TSAT > 25th percentile expressed a negative ranking for stimulated TNF-α and IFN-γ (Th1) responses and positive for IL-6 and IL-10 (Th2) responses, but congenital ID (TSAT ≤25th percentile) did not manifest an overt bias for a predominance of Th2 cytokine responses. However, neonates with congenital ID were more likely to evince positive stimulated TNF-α *Z*-scores. Comparing by sex found no difference between Th1 and Th2 cytokine responses, but the mean Z-scores for both Th1 and Th2 cytokine responses differed in their pattern with positive values for males and, conversely, negative for females, as shown in Figure 2b.

### 3.5. Iron Indices, Inflammation, and CBMC Cytokines by Biological Sex

CB iron indices, inflammatory proteins, and stimulated CBMC cytokine responses by sex are summarized in Table 3. Without considering iron status, few clear sex differences were evident in the hematological and immune profiles. Overall, the CB from males had lower absolute eosinophil counts and were less likely than the females to show signs of eosinophilia (>470/µL) at birth (46% and 54%, respectively, *p* = 0.02). TNF-α and IL-6 levels in the CB plasma from females and males also did not differ. However, following stimulation of the CBMC with PHA, some sex differences were evident, including higher stimulated IFN-γ and IL-6 responses in males when compared to females.

### 3.6. Exploratory Analysis of CBMC Cytokines by Both Iron Status and Biological Sex

In this pilot study, two-way ANOVA modeling was carried out of CBMC IFN-γ and TNF-α cytokine responses based on both ID and sex. Although both were significant, this did not survive correction for multiple comparisons, so we report these as exploratory data, as presented in Table 4. Several sex differences in stimulated CBMC cytokine responses became more evident when considered in the context of the neonate’s iron status. Sex-specific values for the CB plasma cytokines and stimulated cytokine responses subdivided by congenital iron status are provided in Table 4. Stratification by sex revealed that with congenital ID, lower CB plasma IL-6, lower plasma TNF-α levels, and higher stimulated IL-8 responses were specific to females. In contrast, with congenital ID, the higher TNF-α responses after PHA stimulation of the CBMCs were evident in both males and females.

## 4. Discussion

This study of healthy term neonates generated the novel finding that congenital ID can impact the immune responsiveness and cytokine profiles of neonates at delivery. We used CB TSAT to define congenital ID because transferrin receptors are the primary route for mononuclear cell iron entry. In addition, CB plasma ferritin levels were lower in the low TSAT group, consistent with congenital ID. CB hemoglobin and the CB mean cell volume may not fall in congenital ID because more immature cells are larger in maternal obesity ± diabetes [26,28], and inflammation may also prevent the fall in hepcidin. PHA-stimulated CBMC TNF-α and other cytokine responses, and potentially the Th1/Th2 cytokine balance, were also affected in a sexually dimorphic manner. Examining stimulated CBMC cytokine responses is important because cellular reactivity at birth and in early life, including higher TNF-α responses, may be predictive of later atopic disease in childhood [8]; Th1 lymphocytes are vulnerable to iron depletion, especially at birth, and atopic disease can be sexually dimorphic in early life. First, we found that the CBMCs from neonates with congenital ID released more stimulated TNF-α and IL-8 responses than did the CBMCs of IS neonates. The importance of iron status was supported by the in vitro incubation with deferoxamine, which further depleted iron. Considering the whole sample, we found that iron depletion during incubation strengthened the relationships between TNF-α and other cytokines in the NFκβ family after stimulation, a finding anticipated due to the importance of iron in NFκβ regulation. In addition, when stimulated cytokine responses were ranked by Z-score, CBMC Th1 cytokine responses, including TNF-α, were relatively lower than CBMC Th2 cytokine responses in the IS neonates, but Th1-Th2 cytokines did not differ in congenital ID. Further, the effects of congenital ID on cytokine responsiveness differed when demarcated by sex, with lower levels of plasma TNF-α and IL-6, and higher stimulated CBMC IL-8 responses in ID females, but higher CBMC TNF-α responses did not differ by sex in the presence of congenital ID. Iron is required for signaling in the NFκβ family and estrogen may be anti-inflammatory in NFκβ signaling.

Many published studies have found that pediatric undernutrition can adversely affect the immune system. However, only limited published data in human birth cohorts document a link between poor congenital iron status and either infantile atopic disease [3,4,5] or neonatal inflammatory profiles [6]. A potential explanation for a direct effect for iron is that in vitro mechanistic experiments have shown greater vulnerability of Th1 lymphocyte clones to iron depletion than seen for Th2 lymphocytes [25,31]. Studies with animal models also supports that ID could increase the risk of atopic disease. A murine model of atopic asthma indicated that iron supplementation could suppress inflammatory airway responses [32]. In addition, placentas from gravid female rats with ID exhibited higher placental cytokines of TNF-α and IL-6 [33].

One study of human neonates that examined sex found that plasma levels of TNF-α did not differ by sex [34]. However, a different study reported that CB plasma TNF-α levels were lower in males, but that CB plasma levels of IL-6 did not differ by sex [35]. In addition, the CB plasma TNF-α and IL-6 levels were inversely associated with testosterone in males, suggesting that androgens might modulates immune responsiveness in developing male infants [35]. In addition to higher testosterone levels in male fetuses, sex differences could be mediated by the effects of *Sry* gene expression or other sex-related steroid hormones such as DHT [17]. In another large birth cohort, the levels of IFN-γ, IL-5, and IL-10 after CBMC stimulated cytokine responses did not differ by sex [12]. In this pilot study, we could not employ a multivariate model due to the sample size. However, we found in single comparisons that sex-specific differences were evident when cytokine responses were considered with respect to how the balance of Th1 and Th2 cytokines might polarize for a cellular or humoral immune response. Further, another novel finding was that the sex-specific immune responses were modifiable by neonatal iron status. Faster fetal and postnatal growth rates may make male infants more susceptible to congenital and infantile ID [15,36]. These findings also support the previous observation that male fetuses adapt more poorly to intra-uterine stress and may mount less protective immune responses than females [35,37]. In addition, the more robust stimulated CBMC TNF-α and IL-8 responses of the ID females could react differently to antigenic stimuli in the early rearing environment. Taken together, there is a need to systematically interrogate the dual influence of both fetal sex and ID in programming fetal inflammatory responses and creating a predisposition for childhood atopic diseases.

The synthesis of TNF-α, IL-1β, IL-6, IL-8, and IL-12 are regulated by pathways in the NF-κβ family of transcription factors [38]. We found that stimulated CBMC IL-6, IL8, and TNF-α cytokine responses that were differentially affected by iron or revealed the potential interactive effect of iron status and sex were in this NF-κβ family. In support of our findings, deferoxamine affected adult PBMCs through NF-κβ-dependent pathways [31], potentially providing the mechanism accounting for these differences. It is noteworthy that our differentially affected NF-κβ-regulated group of cytokines included both classical Th1 (TNF-α) and Th2 (IL-6) signaling molecules and a more general inflammatory cytokine (IL-8), supporting that iron may have more diverse immune system effects. TNF-α is a major regulator of inflammatory responses. Persistently elevated TNF-α responses and higher plasma TNF-α levels in infancy may be a biomarker predicting persistent wheezing, while higher bronchial lavage TNF-α has been associated with the pathogenesis of asthma in adults [8,39,40]. Lower plasma but higher TNF-α levels in ID could be from separating CBMC from the tonic moderating influence of circulating hormonal and cytokine milieu can promote more robust cytokine responses in culture, as was shown in pregnancy, including inverse relationships [41].

Other findings indicated higher stimulated CBMC IFN-γ responses in males and a remarkable drop in stimulated CBMC IFN-γ responses after chelation. It is plausible that CBMC IFN-γ responses were sensitive to iron depletion due to the role of iron in mitochondrial function, but toxicity is unlikely due to MTT metabolic viability assayed at 94.5% of the PHA values alone. While IFN-γ is typically categorized as a Th1 cytokine polarizing for a cellular immune response, it serves many different immune functions. Previous research has shown that neonatal immunity is immature and cellular immunity as deficient [42]. An atopy-prone neonatal immunophenotype included lower levels of Th1 cytokines, including IFN-γ and IL-12 [43].

Thymocyte populations differentiate and expand at birth, and experiencing ID during this expansion could potentially promote a relative predominance of Th2 clones. At a cellular level, Th1 clonal growth was previously shown to be more vulnerable to iron chelation than that of Th2 clones [31]. Greater PHA-stimulated TNF-α responses in our congenital ID group is in keeping with a recent experiment with monocyte cell lines that showed that mitogen stimulation decreased surface transferrin binding and elevated TNF-α responses, while a preincubation deferoxamine protocol further elevated the TNF-α response [44]. We added deferoxamine simultaneously to CBMC cultures, and as found in our study, simultaneous deferoxamine also blocked the rise in TNF-α [44].

The strengths of our study include utilizing a sensitive, multiplex cytokine array, with few values below the lower limit of detection and a large dynamic range enabling quantification to the ng/mL range for the stimulated cell supernatants. In addition, the CB samples were collected only during the summer months to limit bias due to seasonal variation in allergen exposure. The elapsed time before the collection of cord blood and processing for plasma and cell stimulation was also short when compared to other CB studies [27]. The mitogen PHA was used for cell stimulation because it is traditional stimulant used for CBMC culture [12,27]. But, because other mitogens can polarize the cellular cytokine response differently, it is possible that different findings might be obtained with alternative culture conditions. To minimize the influence of maternal prenatal or perinatal illness, mothers on multiple prescription medications were excluded. Neonatal iron status was defined by TSAT to demarcate groups, because transferrin is the major importer of iron into mononuclear cells. Moreover, this study is one of the few to focus on the possibility of sex-specific nutritional differences in stimulated CBMC cytokine profiles at birth. The study limitations include a moderate sample size, limited power to detect cytokine differences by both ID and sex, and limited racial diversity. The TSAT < 25th percentile of our cohort (TSAT < 36%) included 60% obese mothers, which represents a risk for congenital ID. However, based on iron accretion and published CB population norms, a TSAT < 36% approximates the 5th percentile [45]. Notwithstanding the occurrence of low iron levels in a subset of the infants, our relatively healthy maternal and neonatal cohort did not fully represent the full range of the clinical population in obstetrical practices. However, the focus on elective cesarean delivery did confer the advantage of reducing the variability of the CB plasma cytokines [46], because higher cord levels of both IL-1 and TNF-α are seen in chorioamnionitis and long, difficult labors may impact tissue IL-6 levels [47]. In addition, we did not have the maternal or paternal history of atopic disease, so did not assess that contribution to the findings. In addition, we previously showed that Class II obesity in women can adversely affect the iron status and cytokine responses of neonates at delivery [26]; therefore, we analyzed maternal obesity and diabetes, but these were not determined to be confounding variables in our study.

## 5. Conclusions

**Figure 3:**
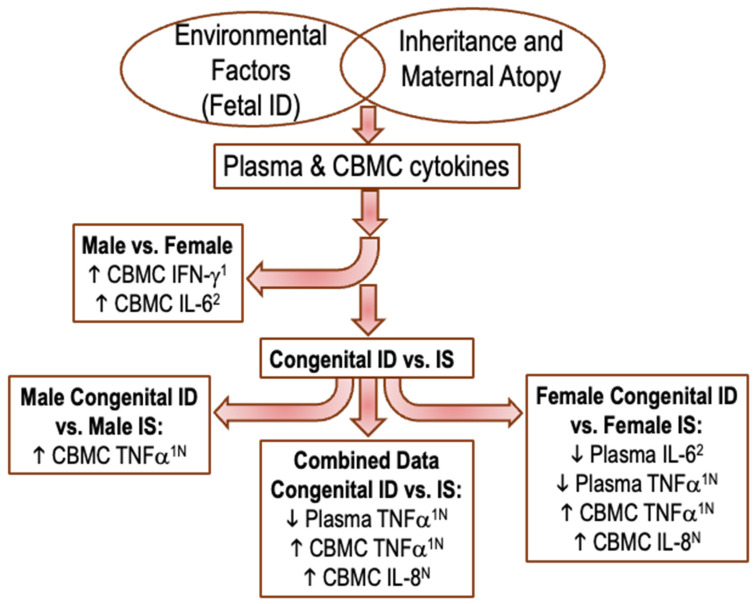
Conceptual diagram describing findings. Environmental factors such as congenital ID in left oval overlap with inheritance and maternal atopy factors in right oval to impact plasma and stimulated CBMC cytokines. Biological sex itself impacted stimulated CBMC responses, middle left. In congenital ID vs. IS (bottom), significant male stimulated CBMC response are represented on the left, significant plasma and stimulated CBMC responses with both sexes combined are represented in the center, and significant female congenital ID plasma cytokines and stimulated CBMC responses are represented on the right. Arrows, direction of cytokine; ^1^, Th1 cytokines; ^2^, Th2 cytokines; ^N^, the nuclear factor-κβ (NF-κβ)-dependent family member.

Although our study should be considered exploratory because of the moderate sample size, the numbers were sufficient to identify neonatal iron status as an important factor that can influence the immune responsiveness of neonates in a way that could predispose for atopic disease. A summary conceptual diagram is provided in Figure 3. Because the CB samples were de-identified and obtained from a representative healthy population, it was not possible to conduct serial assessments, so it will be important to replicate or extend this study as a prospective, longitudinal study. Based on our results, it will be critical to consider the possibility that the immune effects of being iron deficient manifest differently in female and male infants. This is important because male fetuses and infants have higher proinflammatory cytokine production and IgE levels than females, but female infants express different toll-like receptors and respond to immunizations better than males. Many of these differences persist into late childhood and adulthood [48]. Research with animal models continues to highlight the complex processes that underlie sex-based differences in immunity, including acute and long-term influences on prenatal hormones. Our findings are in keeping with many studies that identified the early developmental origins of atopic diseases, with an emphasis on the prenatal processes that shape how the maturing infant responds to the antigenic and infectious factors in the rearing environment.

## Figures and Tables

**Table 1:** Demographic Characteristics.

Ethnic and Racial Distribution	
Caucasian	73.0%
African American	5%
Hispanic/Latina	11%
Asian/Southeast Asian	11%
Insurance coverage	
Medicaid insurance	27%
Health maintenance organization or insurance	73%
Maternal obesity body mass index > 30 kg/m^2^	60%
Maternal diabetes	13%
Maternal diabetes by body mass index (BMI)	
If BMI < 30 kg/m^2^	0%
If BMI ≥ 30 but <35 kg/m^2^	25%
If BMI ≥ 35 kg/m^2^	37.5%
Any maternal hypertension	0%
Newborn sex	
Female	47%
Male	44%
Gestational size	
Appropriate for Gestational Age (AGA)	78%
Small for Gestational Age (SGA)	11%
Large for Gestational Age (LGA)	11%
NICU admission	0%
High-risk (≥3) ID risk factors	30%

**Table 2:** CB iron indices, inflammatory markers, and PHA and deferoxamine-stimulated CBMC cytokine responses by iron status.

Iron Indices, and Blood and Plasma Inflammatory Markers	TSAT > 25th Percentile IS (N = 59)	TSAT ≤ 25th Percentile ID (N = 26)	*p*-Value
Hemoglobin (g/L)	146 (13)	150 (14)	0.262
Mean cell volume (fL)	107.7 (5.0)	106.9 (5.2)	0.617
Zinc protoporphyrin/heme (µmol/mol) ^a^	100.0 (39.0)	114.0 (34.0)	0.399
Plasma ferritin (µg/L)	96.1(51)	75.7 (41.4)	0.025
Plasma hepcidin (ng/mL)	14.7 (5.7)	14.7 (7.6)	0.982
White blood cells (cells × 10^9^/L)	13.0 (3.7)	12.3 (2.9)	0.535
Lymphocyte (10^9^/L)	4.85 (1.10)	5.06 (1.43)	0.719
Eosinophils (10^9^/L)	0.4 (0.3)	0.4 (0.2)	0.624
Plasma C-reactive protein (mg/L)	6.7 (5.9)	4.5 (4.4)	0.156
Plasma IL-6 (pg/mL)	1.7 (1.2)	1.9 (1.9)	0.592
Plasma tumor necrosis factor (TNF)-α (pg/mL)	1.7 (0.4)	1.4 (0.4)	0.017
PHA-stimulated CBMC cytokines			
IL-1β (pg/mL) ^a^	1470 (2331)	1007 (1759)	0.132
IL-5 (pg/mL)	12.5 (12.5)	20.7 (25.2)	0.421
IL-6 (pg/mL) ^a^	3407 (873)	3563 (2671)	0.677
IL-8 (pg/mL) ^a^	3528 (2630)	6304 (3241)	0.007
IL-10 (pg/mL)	340.6 (273.0)	345.4 (255.8)	0.963
IL-12 (pg/mL)	115.9 (48.5)	108.1 (35.8)	0.661
Tumor necrosis factor (TNF)-α (pg/mL) ^a^	2760.7 (816.9)	4554.8 (4159.2)	0.013
Interferon (IFN)-γ (pg/mL)	276.3 (154.8)	400.1 (290.4)	0.054
PHA-plus-deferoxamine-stimulated CBMC cytokines			
IL-1β (pg/mL) ^a^ **↑↑**	2691 (1452)	2854 (2730)	0.790
IL-5 (pg/mL) **↑↑**	94.9 (19.7)	122.9 (58.5)	0.570
IL-6 (pg/mL) ^a^ **↔↔**	3354 (633)	3197 (2836)	0.432
IL-8 (pg/mL) ^a^ **↔↔**	3245 (2798)	5674 (3967)	0.132
IL-10 (pg/mL) **↓↓**	5.4 (0.1)	5.0 (0.3)	0.153
IL-12 (pg/mL) ^a^ **↔↔**	106.1 (59.7)	98.7 (19.4)	0.360
Tumor necrosis factor (TNF)-α (pg/mL) ^a^ **↔↓**	2797 (602)	2662 (2064)	0.032
Interferon (IFN)-γ (pg/mL) **↓↓**	5.6 (0.1)	5.2 (0.2)	0.049

IS, iron-sufficient; ID, iron-deficient; CBMC, cord blood mononuclear cell; IL, interleukin; PHA, phytohemagglutinin; TSAT, transferrin saturation; IFN-γ, interferon gamma. Arrows show direction of deferoxamine effect on CBMCs vs. PHA only, **↓↓** both IS and ID >10% lower than PHA; **↑↑** both IS and ID >10% higher than PHA; **↔↔** both IS and ID within 10% of PHA; **↔↓** IS was unchanged for TNF-α, but ID was >10% lower. Data presented as mean (SD) or ^a^ median (IQR). The 25th percentile value for TSAT is 36%.

**Table 3:** Cord blood iron indices, inflammatory indices, and PHA-stimulated CBMC cytokines by biological sex.

Iron Indices, and Blood and Plasma Inflammatory Markers	Female (N = 39)	Male (N = 38)	*p*-Value
Hemoglobin (g/L)	147 (13)	147 (16)	0.935
Mean cell volume (fL)	108.0 (4.3)	105.8 (5.2)	0.049
Zinc protoporphyrin/heme (µmol/mol) ^a^	104.5 (41.0)	100.0 (39.5)	0.519
Plasma ferritin (µg/L)	78.6 (39.3)	83.6 (54.1)	0.827
Plasma hepcidin (ng/mL)	14.7 (5.6)	13.4 (5.9)	0.354
WBCs (cells × 10^9^/L)	13.1 (3.0)	12.1 (3.4)	0.156
Lymphocytes (10^9^/L)	5.13 (1.34)	4.95 (1.43)	0.454
Eosinophils (10^9^/L)	0.5 (0.3)	0.4 (0.2)	0.043
Plasma C-reactive protein (mg/L)	5.7 (7.0)	6.2 (6.2)	0.743
Plasma IL-6 (pg/mL)	1.6 (1.1)	1.9 (1.5)	0.486
Plasma tumor necrosis factor (TNF)-α (pg/mL)	1.6 (0.3)	1.6 (0.5)	0.81
PHA-stimulated CBMC cytokines			
IL-1β (pg/mL) ^a^	1192 (1945)	1149 (24,442)	0.702
IL-5 (pg/mL)	14.9 (18.0)	15.7 (16.3)	0.407
IL-6 (pg/mL) ^a^	3307 (541)	3639 (2436)	0.03
IL-8 (pg/mL) ^a^	3334 (2365)	3539 (2991)	0.146
IL-10 (pg/mL)	325.6 (252.8)	294.7 (187.5)	0.73
IL-12 (pg/mL)	103.6 (43.6)	120.6 (43.1)	0.052
Tumor necrosis factor (TNF)-α (pg/mL) ^a^	2750 (1230)	3000 (2091)	0.087
Interferon (IFN)-γ (pg/mL)	318.8 (314.6)	422.3 (284.9)	0.02

CBMC, cord blood mononuclear cell; IL, interleukin; PHA, phytohemagglutinin; WBCs, white blood cells. Data presented as mean (SD) or median (IQR); ^a^, indicates median (IQR).

**Table 4:** Cord blood inflammatory markers and PHA-stimulated CBMC cytokines by transferrin saturation stratified by biological sex.

	Male			Female		
	TSAT > 25th Percentile	TSAT ≤ 25th Percentile	*p*-Value	TSAT > 25th Percentile	TSAT ≤ 25th Percentile	*p*-Value
	IS N = 27	ID N = 11		IS N = 29	ID N = 10	
Inflammatory markers						
WBCs (cells × 10^3^/µL)	13.1 (3.7)	12.3 (1.8)	0.998	12.65 (4.11)	12.66 (3.61)	0.571
Lymphocytes (/µL)	5.02 (1.18)	5.11 (1.83)	0.908	4.75 (1.12)	4.84 (0.80)	0.76
Eosinophils (/µL)	0.4 (0.3)	0.3 (0.2)	0.983	0.48 (0.34)	0.39 (0.18)	0.844
Plasma CRP (mg/L)	8.8 (8.7)	3.4 (2.3)	0.129	4.74 (2.58)	6.81 (6.22)	0.232
Plasma IL-6 (pg/mL)	1.6 (0.7)	2.4 (2.4)	0.632	1.66 (0.74)	0.78 (0.30)	0.003
Plasma TNF-α (pg/mL)	1.6 (0.4)	1.2 (0.6)	0.131	1.62 (0.29)	1.28 (0.29)	0.02
PHA-stimulated CBMC cytokines and cytokine ratios						
IL-1β (pg/mL) ^a^	2996 (1979)	1666 (993)	0.93	1646 (1244	1550 (930)	0.468
IL-5 (pg/mL)	11.1 (7.1)	18.0 (18.0)	0.404	13.7 (6.9)	23.9 (13.1)	0.51
IL-6 (pg/mL) ^a^	2572 (1694)	2887 (2411)	0.926	387 (1378)	4072 (2477)	0.276
IL-8 (pg/mL) ^a^	2477 (1484)	402.1 (1503)	0.14	608 (1167)	4546 (2036)	0.034
IL-10 (pg/mL)	308.2 (263.4)	356.4 (343.3)	0.966	316.1 (228.1)	428.9 (436.3)	0.278
IL-12 (pg/mL)	131.9 (127.8)	128.9 (121.5)	0.974	103.6 (95.7)	109.2 (109.2)	0.487
TNF-α (pg/mL) ^a^	1471 (1158)	3327 (2551)	0.033	1633 (1331)	2354 (5148)	0.046
IFN-γ (pg/mL)	327.5 (295.6)	507.3 (331.9)	0.449	236.0 (200.1)	366.95 (346.42)	0.046

IS, iron sufficient; ID, iron deficient; CRP, C-reactive protein; CBMC, cord blood mononuclear cell, IL, interleukin; PHA, phytohemagglutinin; TNF-α, tumor necrosis factor alpha; IFN-γ, interferon gamma; TSAT, transferrin saturation; WBCs, white blood cells. Data presented as mean (SD) or ^a^ median (IQR). The 25th percentile value for TSAT is 36%.

## Data Availability

All data retained during the study are de-identified and will be shared with anyone requesting data access for any research purpose. The data will be available from the corresponding author, Dr. Kling, pkling@wisc.edu, immediately following publication.
